# Urinary Biomarkers of Phthalates Exposure, Blood Lead Levels, and Risks of Thyroid Nodules

**DOI:** 10.3390/toxics9030068

**Published:** 2021-03-22

**Authors:** Jingsi Chen, Yi Chen, Shaojie Liu, Bo Chen, Yingli Lu, Ruihua Dong

**Affiliations:** 1 Department of Nutrition and Food Hygiene, School of Public Health, Soochow University, Suzhou 215123, China; 15211020022@fudan.edu.cn; 2 Key Laboratory of Public Health Safety of Ministry of Education, Collaborative Innovation Center of Social Risks Governance in Health, School of Public Health, Fudan University, Shanghai 200032, China; 18211020023@fudan.edu.cn (S.L.); chenb@fudan.edu.cn (B.C.); 3 Institute and Department of Endocrinology and Metabolism, Shanghai Ninth People’s Hospital, Shanghai Jiao Tong University School of Medicine, Shanghai 200011, China; chenyi9h@126.com

**Keywords:** case-control study, thyroid nodules, phthalates, blood lead levels, moderation effect

## Abstract

Thyroid nodules (TNs) are becoming increasingly prevalent. However, few studies have reported the effects of phthalates and lead (Pb) on TNs. In this study, we aimed to explore the associations of phthalates and Pb with the risks of TN. We sex-age-matched 220 TNs patients and 220 healthy controls from Zhejiang Shangyu, China. We measured 13 phthalate metabolites in spot urine samples. Blood lead levels (BLLs) were determined by atomic absorption spectrometry. The multivariable logistic regression models were used to assess the associations between urinary phthalate metabolites and BLLs and the risks of TNs. We found BLLs were associated with increased risk of TNs in total population. Female-specific positive associations of mono-2-ethyl-5-carboxypentylphthalate (MECPP), mono-2-ethyl-5-hydroxyhexylphthalate (MEHHP), mono-2-ethyl-5-oxohexylphthalate (MEOHP), mono-2-carboxymethyl-hexyl phthalate (MCMHP), and mono-isononyl phthalate (MiNP) with increased risk of TNs were also observed. Moreover, the positive association between phthalates and TNs was modified by BLLs. At the highest tertile of BLLs, monoethylphthalate (MEP), MECPP, MEHHP, MEOHP, and MiNP were significantly associated with increased risk of TNs. Our results indicated that certain phthalate metabolites and BLLs may contribute to increased risks of TNs.

## 1. Introduction

Thyroid nodules (TNs) are becoming increasingly prevalent [1]. TNs may result in a variety of clinical sequelae, especially thyroid cancer [2,3]. China had high prevalence of TNs as being estimated to be 30–50% in healthy adults [4,5]. Women were reported to be more frequently affected than men [6]. The high prevalence of TNs may be attributed to the advancements in diagnostic technologies. However, accumulating evidence in recent years suggested that exposure to some endocrine chemicals might be also responsible for the increasing incidence of TNs [7].

One type of endocrine disruptor is phthalates, which have been reported to disrupt thyroid function and its growth. Several rodent experiments indicated that phthalates exposure might adversely influence thyroid homeostasis [8]. The epidemiologic data also reported that phthalates may adversely influence thyroid hormone levels among different populations including infants, adolescents, and adults [9,10,11,12]. Phthalates exposure alters thyroid hormone levels and disrupts thyroid hormone homeostasis, which may be responsible for TNs [13,14,15]. Nevertheless, the human data are scarce regarding the effects of phthalates on TNs.

Lead (Pb) is another chemical reported to impact thyroid function [16,17]. Several epidemiology studies reported the association between Pb exposure and thyroid hormone levels. For example, Dundar et al. (2006) found that serum Pb could significantly reduce free thyroxine (FT4) levels level in adolescents [18]. Maryam found that Pb can increase the risk of developing hypothyroidism and thyroid cancer [19]. However, the human evidence is also scarce and inconsistent. Moreover, there is no human study concerned the effect of Pb on TNs.

Both phthalates and Pb are common chemicals that are commonly used in daily consumer products, resulting in ubiquitous human exposure [20,21]. In this study, we conducted a case–control study to explore the associations of phthalates and Pb exposure as well as their interaction with the risk of having TNs.

## 2. Materials and Methods

### 2.1. Study Participants and Data Collection

All participants were a subset of Survey on Prevalence in East China for Metabolic Diseases and Risk Factors (SPECT China). More detailed sampling information is published in previous articles [22]. For this study, 628 subjects in Shangyu city of Zhejiang province who provided urine samples were recruited. We excluded the participants who had a history of thyroid surgery (*n* = 14), thyroid diseases (*n* = 8), and missing thyroid ultrasound (US) information (*n* = 5). Two-hundred-and-twenty participants who were diagnosed with TNs (multiple nodules or single nodule with any lesion ≥2 mm in diameter) by ultrasound were included into TNs group. The sex-age-matched (±3 years) control group (*n* = 220) was recruited from healthy participants of the aforementioned Shangyu sample set. All participants were administered with a questionnaire interview to collect basic demographics characteristics and lifestyle, medical, and family history. They also received comprehensive physical and biochemical examinations and B-mode ultrasonic examinations. The detailed information of thyroid examinations and the definition of thyroid nodule were published in previous articles [23].

In addition, all participants had their blood lead levels (BLLs) measured. BLLs were determined by atomic absorption spectrometry (BH2200, China) [24]. In brief, 0.1 mL blood sample was thawed, transferred to a 1.5 mL plastic centrifuge tube, and mixed with 0.9 mL Triton solution (0.1%). Then, the mixture was analyzed by atomic absorption spectrometry. The average recoveries and relative standard deviations (RSD) were 98.7% and 0.9% at 40 μg/dL, 104.1%, and 2.5% at 1 μg/dL. The limits of detection (LOD) was 0.6 μg/dL.

All individuals provided informed consent prior to data collection. The study protocol was approved by the ethics committee of the Shanghai Ninth People’s Hospital, Shanghai Jiao Tong University School of Medicine.

### 2.2. Measurement of Urinary Metabolites of Phthalates

We used the method of ultra-performance liquid chromatography tandem mass spectrometry (UPLC-MS/MS) to detect 13 urinary metabolites of phthalates, including monomethyl phthalate (MMP), monoethylphthalate (MEP), mono-n-butylphthalate (MnBP), monoisobutylphthalate (MiBP), mono-benzylphthalate (MBzP), mono-2-ethylhexylphthalate (MEHP), mono-2-ethyl-5-oxohexylphthalate (MEOHP), mono-2-ethyl-5-hydroxyhexylphthalate (MEHHP), mono-2-ethyl-5-carboxypentylphthalate (MECPP), mono-2-carboxymethyl-hexyl phthalate (MCMHP), mono-(3-carboxypropyl) phthalate (MCPP), mono-isononyl phthalate (MiNP), and mono-cyclohexyl phthalate (MCHP).

The method has been described in our previous study with slight modification [25]. Briefly, we thawed 1 mL of urine sample, transferred it to a 10 mL glass tube, and incubated with β-glucuronidase (*E. coli* K 12; Roche, Mannheim, Germany) at 37 °C for 120 min. Subsequently, the sample was mixed with 1 mL of aqueous 2% (*v*/*v*) acetic acid and 100 μL of internal standard (100 μg/L). The mixture was loaded into a PLS column (Dikma, China; 60 mg/3 mL) previously activated with 2 mL methanol and 2 mL of aqueous 0.5% (*v*/*v*) acetic acid. After sample loading, the column was washed with 2 mL of aqueous 0.5% (*v*/*v*) acetic acid. Next, 1 mL of methanol was added to elute metabolites. Finally, the eluate was passed through a 0.2 μm filter and analyzed (2 μL) by an UPLC-MS/MS system integrated by Waters ACQUITY UPLC H-Class (Waters, Milford, MA, USA) coupled with an ABSCIEX QTRAP 6500 (AB Sciex Technologies, Framingham, MA, USA). The analytical column was a Waters ACQUITY UPLC BEH C18 Column (1.7 µm, 2.1 × 50 mm, Waters, Milford, MA, USA).

An internal standard method was used to quantify the target metabolite. For every 20 samples, a procedural blank and two matrix-spiked samples at two different spiking concentrations (5 and 15 ng/mL) were processed. The average recoveries and RSD of target metabolites ranged from 86.5% to 123.1% and from 0.5% to 12.5% at 5 μg/L, and ranged from 75.3% to 98.4% and from 0.7% to 8.8% at 15 μg/L, respectively. Sample concentrations of these metabolites were determined after subtraction of blank values. The LOD was calculated at signal-to-noise (S/N) of 3, with concentration of 0.100, 0.080, 0.006, 0.006, 0.060, 0.006, 0.010, 0.020, 0.020, 0.040,0.100, 0.010, and 0.004 μg/L for MMP, MEP, MnBP, MiBP, MBzP, MEHP, MEOHP, MEHHP, MECPP, MCMHP, MCPP, MiNP, and MCHP, respectively.

### 2.3. Statistical Analysis

Data analyses were performed using IBM SPSS Statistics. *p* values are presented as two-tailed with a significance level of 0.05. The differences in demographic characteristics, urinary phthalate metabolites, and BLLs between case and control subjects were analyzed using Mann-Whitney U test, Student t test, and χ^2^ test depending on the distribution of the data. The *p* values were adjusted using false discovery rate. We conducted multiple logistic regression analysis to assess the associations of urinary phthalate metabolites and BLLs with risk of TNs. Urinary phthalate metabolites and BLLs were modeled as continuous variables in the logistic models to calculate the odds ratios (ORs) for TNs. We adjusted several potential confounders including age, sex, BMI, current smoking status, current alcohol status and educational level in the regression models. Because the incidence of TNs has a gender disparity, we further analyzed the gender difference between the urinary phthalate metabolites, BLLs, and the risks of TNs. Furthermore, we analyzed the effect modification by BLLs between the urinary phthalate metabolites and the risk of having TNs. The BLLs were categorized into tertiles. The categorical BLLs and urinary phthalate metabolites were included into the models as interaction terms.

## 3. Result

### 3.1. Population Characteristics

The demographic information of the 418 individuals is summarized in Table 1. No differences were found between TNs patients and the controls. Concentration distribution of BLLs and urinary metabolites of phthalates are listed in Table 2. TNs patients and controls had no difference in the concentrations of phthalates metabolites and BLL.

### 3.2. Regression Results

The associations between urinary phthalate metabolites, BLLs, and risk of TNs in the overall population are presented in Table 3. We found that BLLs were associated with increased risk of TNs. Table 4 presents the associations stratified by gender. Increased risks of TNs were only observed among females. MECPP, MEHHP, MEOHP, MCMHP, and MINP were significantly associated with increased risk of TNs in female.

### 3.3. Evaluation of Effect Modification

We found effect modification by BLLs between phthalate metabolites and risks of TNs among female but not male (Table 5, data are not shown for males). At the second and third tertile of BLLs, MEP, MECPP, MEHHP, MEOHP, and MINP were significantly associated with increased risk of TNs compared to the lowest tertile of BLLs.

## 4. Discussions

In this study, we found some urinary phthalate metabolites and BLLs increased the risk of TNs. We also observed some sex-specific associations of specific urinary phthalate metabolites with the risk of TNs. Third, we observed effect modification by BLLs between phthalate metabolites and risk of TNs among females but not males.

Some studies have observed the association between phthalates exposure with thyroid disorders [9,10,26]. Although TNs had high prevalence in the general population, there was few epidemiological studies examining the relationships between phthalates with TNs. To our knowledge, there were only two recent studies examined the association between phthalates and the risks of TNs. For example, one study detected DEHP and MEHP concentrations in 27 TNs and 28 thyroid cancer patients, and this study found exposure to DEHP metabolites in TNs patients increases susceptibility to develop thyroid cancer [27]. Another case-control study sex-matched 138 TN patients, 144 thyroid cancer, and 144 healthy controls from China. They also found the exposure of DEHP metabolites positively associated with risks of TNs. However, MBP exposure decreased the risks of TNs and thyroid cancer [28]. Furthermore, this study found male-specific positive associations between specific phthalates exposure and the risk of thyroid cancer. On the contrary, we found female-specific positive associations between specific phthalate metabolites and the risk of TNs.

The disparate findings by previous study compared to our study may be attributable to the different population, as well as to different exposure levels to phthalates. Phthalates are widely used as plasticizers in cosmetics, food packaging, and other industrial products. Due to the reversible combination to plastic matrix, phthalates can leach out from the plastics and population may be exposed to phthalates through application of cosmetics, the ingestion of food and drinking water, and inhalation of indoor and outdoor air. As with different lifestyles, the different populations had different exposure levels to phthalates. For example, except for MMP and MEHP, the urinary concentration of other metabolites observed by Liu. et al. was higher than that in our study. MEP in Liu et al. (13.02 μg/g) was 2 times higher than that detected in this study (7.35 μg/g). Note that because of the very small sample sizes of males (*n* = 40) in the study conducted by Liu et al., more human studies are needed to identify the associations of the exposure to phthalates with the risks of TNs, as well as the sex difference.

Several mechanisms may explain the association of phthalates with increased TNs risk. First, previous studies found relationships of urinary phthalate metabolites with oxidative stress in different populations [29,30,31]. Thus, exposure to certain phthalates induce oxidative stress in thyroid which could impact the development and progression of TNs. Second, phthalates exposure was found to disrupt thyroid hormone homeostasis [13,32]. Third, altered thyroid hormone levels, and disrupted hypothalamus-pituitary-thyroid (HPT) axis, may also attribute to the development and progression of TNs [13,33].

A few studies have explored the relationship between BLLs and thyroid hormones, while most of them were conducted in small occupational populations [34]. In general, the BLLs in controls of our study was higher, but comparable with the levels reported in general population of China [35,36] and in several U.S. NHANES studies [37,38]. According to the recent definitions by the U.S. Centers for Disease Control and Prevention and National Institute of Occupational Safety and Health (NIOSH), BLLs greater than or equal to 5 μg/dL are considered as to be elevated in adults [39]. The median BLLs for our study participants was 3.7 μg/dL, which was lower than 5 μg/dL. Although the toxic effects of Pb at high level on thyroid function have been reported, the associations between “relatively lower level” of Pb and thyroid function in general populations are still scarce. To the best of our knowledge, this is the first study to explore associations of Pb with TNs in the general population. A recent study reported that higher content of Pb in the thyroid gland could be involved in the etiology of multinodular goiter disease [17]. Although the underlying mechanisms are still unclear, altered thyroid hormone levels and disrupted immune system may play a role in the development and progression of TNs [34].

As mentioned, phthalates and Pb disrupt thyroid function through different mechanisms. Therefore, it is not unexpected that we first explored effect modification by BLLs between phthalate metabolites and risks of TNs. There are several potential mechanisms by which phthalates and Pb may interact to affect thyroid function. On the one hand, both phthalates and Pb were positively related to TSH [34]. The rate of increased serum TSH was positively associated with the rate of the malignant nature of TNs [40,41]. On the other hand, both phthalates and Pb may modulate the immune system and alter circulating markers of inflammation [34,42]. Our results highlight the roles of phthalates and Pb in the largely unclarified etiology of TNs. Therefore, more studies are needed to reveal the potential mechanisms.

Our study has several shortcomings. First, one-spot urine was not enough to present the exposure level of phthalates because of the short half-lives of phthalates. Second, blood Pb, which mostly reflects relatively recent exposure, is an inadequate method to measure the body burden of Pb. Second, we did not take into account confounders such as the consumption of lithium, which may need to be considered. Third, the cross-sectional, case–control design could not adequately control for significant potential confounders, and limits our ability to explore the exposure–outcome associations between phthalates, Pb and TN. Finally, the cross-sectional design is not appropriate to allow the causal interpretation of the findings.

## 5. Conclusions

We observed increased risks of TNs for specific urinary phthalate metabolites and BLLs. Moreover, some specific phthalate metabolites increased risks of TNs only in females but not males. Finally, we found the positive associations between phthalates and TNs were modified by BLLs among females.

## Figures and Tables

**Table 1:** Characteristics of thyroid nodule and corresponding health controls (*n* = 440).

Characteristic	Cases (*n* = 220)	Controls (*n* = 220)	*p*-Values
Sex, n (%)			1.000
Male	101 (45.9)	101 (45.9)	
Female	119 (54.1)	119 (54.1)	
Age, median (IQR), years	62 (54, 69)	62 (53, 69)	0.912
BMI, kg/m^2^	23.5 (21.5, 25.8)	23.6 (21.0, 25.7)	0.340
Education, n (%)			
≤Primary school	37 (16.8)	39 (17.7)	0.931
High school or technical Secondary school	160 (72.7)	172 (78.2)	
≥College graduate	23 (10.5)	9 (4.1)	
Smoke, n (%)			0.829
Yes	52 (23.6)	50 (22.7)	
No	142 (64.5)	149 (67.7)	
Quit	26 (11.8)	21 (9.6)	
Alcohol consumpution, n (%)			0.673
Current drinker	88 (40.0)	92 (41.8)	
Non-drinker	115 (52.3)	112 (50.9)	
Former drinker	17 (7.7)	16 (7.3)	
Urinary iodine, median (IQR), μg/L	141.3 (84.3, 205.2)	148.8 (97.0, 199.8)	0.462

IQR: interquartile range.

**Table 2:** Urinary concentrations of phthalate metabolites (μg/g) and blood lead levels (μg/dL) in study population.

Concentrations	Cases (*n* = 220)	Controls (*n* = 220)	*p*-Adjusted Values
MMP	3.14 (1.38, 8.49)	3.20 (1.64, 6.27)	0.815
MEP	6.04 (2.4, 18.91)	7.28 (2.76, 24.25)	0.815
MiBP	9.58 (4.01, 23.94)	10.21 (4.63, 23.53)	0.815
MnBP	9.01 (2.3, 25.25)	10.87 (1.66, 26.38)	0.815
MBzP	0.40 (0.23, 0.89)	0.44 (0.23, 0.69)	0.815
MEHP	2.87 (0.98, 37.76)	2.66 (1.00, 31.99)	0.985
MECPP	7.3 (3.74, 15.33)	6.39 (3.42, 11.22)	0.815
MEHHP	7.59 (3.17, 19.85)	6.56 (2.87, 13.48)	0.495
MEOHP	3.76 (1.99, 9.25)	3.46 (1.70, 6.32)	0.787
MCMHP	0.67 (0.26, 3.7)	0.65 (0.21, 1.93)	0.787
MCPP	1.93 (1.18, 3.53)	1.77 (1.12, 2.97)	0.815
MiNP	8.13 (4.02, 18.8)	7.49 (3.77, 13.87)	0.815
MCHP	0.03 (0.02, 0.06)	0.02 (0.01, 0.04)	0.495
BLLs	4.00 (3.00, 5.30)	3.60 (2.70, 4.88)	0.322

*p*-adjusted values: *p*-values between control and thyroid nodule group, adjusted using false discovery rate.

**Table 3:** Adjusted ORs for urinary phthalate metabolites, blood lead levels, and thyroid nodule.

Metabolites	Total Population	
	OR (95% CI) ^a^	*p*-Values
MMP	0.96 (0.80, 1.15)	0.663
MEP	0.95 (0.85, 1.06)	0.324
MiBP	0.96 (0.87, 1.05)	0.370
MnBP	0.98 (0.92, 1.03)	0.412
MBzP	0.90 (0.72, 1.13)	0.347
MEHP	0.97 (0.89, 1.04)	0.387
MECPP	1.11 (0.92, 1.34)	0.290
MEHHP	1.07 (0.95, 1.21)	0.277
MEOHP	1.01 (0.88, 1.17)	0.851
MCMHP	1.08 (0.96, 1.22)	0.219
MCPP	0.96 (0.73, 1.26)	0.766
MiNP	1.09 (0.92, 1.29)	0.305
MCHP	1.16 (0.90, 1.50)	0.251
BLLs	1.10 (1.01, 1.19)	0.028 *

^a^ The model was constructed with adjusting age, sex, BMI, smoking status, drink status, education level, and urinary iodine. Bold italic *: *p* < 0.05; ORs: odds ratios.

**Table 4:** Adjusted ORs for urinary phthalate metabolites, blood lead levels, and thyroid nodule stratified by gender.

Metabolites	Female		Male	
	OR (95% CI) ^a^	*p*-Values	OR (95% CI)	*p*-Values
MMP	1.17 (0.91, 1.50)	0.235	0.77 (0.59, 1.01)	0.061
MEP	1.10 (0.95, 1.27)	0.218	0.79 (0.66, 0.93)	***0.006*** *
MiBP	1.03 (0.90, 1.17)	0.662	0.86 (0.74, 1.01)	0.070
MnBP	1.03 (0.95, 1.11)	0.497	0.93 (0.82, 1.04)	0.083
MBzP	1.26 (0.89, 1.78)	0.190	0.68 (0.50, 0.94)	***0.018*** *
MEHP	0.96 (0.86, 1.07)	0.470	0.97 (0.87, 1.09)	0.623
MECPP	1.36 (1.02, 1.81)	***0.034*** *	0.92 (0.70, 1.20)	0.532
MEHHP	1.22 (1.02, 1.47)	***0.030*** *	0.93 (0.77, 1.12)	0.433
MEOHP	1.38 (1.07, 1.78)	***0.015*** *	0.85 (0.71, 1.03)	0.103
MCMHP	1.21 (1.02, 1.45)	***0.034*** *	0.97 (0.81, 1.15)	0.691
MCPP	1.01 (0.68, 1.49)	0.957	0.92 (0.63, 1.33)	0.647
MiNP	1.39 (1.04, 1.86)	***0.026*** *	0.92 (0.73, 1.15)	0.472
MCHP	1.34 (0.92, 1.96)	0.126	1.03 (0.72, 1.45)	0.888
BLLs	1.01 (1.00, 1.02)	0.056	1.01 (1.00, 1.03)	0.055

^a^ The model was constructed with adjusting age, sex, BMI, smoking status, drink status, education level, and urinary iodine. Bold italic *: *p* < 0.05; ORs: odds ratios.

**Table 5:** ORs for the association between urinary phthalate metabolites and thyroid nodule according to blood lead levels in female.

Metabolites	Q1	Q2		Q3		
		OR (95% CI) ^a^	*p*-Values	OR (95% CI)	*p*-Values	*p*-Interaction
MMP	1	1.32 (0.97, 1.80)	0.075	1.30 (0.86, 1.98)	0.215	0.090
MEP	1	1.21 (0.95, 1.56)	0.120	1.38 (1.08, 1.75)	0.010 *	0.018 *
MiBP	1	1.12 (0.94, 1.33)	0.216	1.16 (0.93, 1.44)	0.184	0.224
MnBP	1	1.11 (0.98, 1.25)	0.119	0.97 (0.84, 1.11)	0.608	0.611
MBzP	1	0.89 (0.56, 1.41)	0.617	0.82 (0.49, 1.37)	0.444	0.950
MEHP	1	0.97 (0.82, 1.16)	0.752	1.04 (0.87, 1.25)	0.653	0.865
MECPP	1	1.38 (1.05, 1.81)	0.022 *	1.44 (1.04, 2.00)	0.027 *	0.007 *
MEHHP	1	1.33 (1.04, 1.70)	0.024 *	1.21 (0.93, 1.56)	0.152	0.025 *
MEOHP	1	1.36 (1.03, 1.80)	0.030 *	1.46 (1.07, 2.00)	0.017 *	0.013 *
MCMHP	1	1.18 (0.89, 1.57)	0.255	1.18 (0.83, 1.66)	0.357	0.065
MCPP	1	1.39 (0.79, 2.47)	0.258	1.09 (0.57, 2.10)	0.786	0.652
MiNP	1	1.33 (1.02, 1.73)	0.034 *	1.51 (1.11, 2.06)	0.009 *	0.003 *
MCHP	1	0.86 (0.71, 1.05)	0.137	0.84 (0.67, 1.02)	0.074	0.180

^a^ The model was constructed with adjusting age, sex, BMI, smoking status, drink status, education level, and urinary iodine. Bold italic *: *p* < 0.05.

## Data Availability

The data presented in this study are available on request from the corresponding author. The data are not publicly available due to them containing information that could compromise research participant privacy.
